# Simulation of Regionally Ecological Land Based on a Cellular Automation Model: A Case Study of Beijing, China

**DOI:** 10.3390/ijerph9082986

**Published:** 2012-08-17

**Authors:** Hualin Xie, Chih-Chun Kung, Yanting Zhang, Xiubin Li

**Affiliations:** 1 Institute of Poyang Lake Eco-economics, Jiangxi University of Finance and Economics, Nanchang 330013, China; Email: cckung78@hotmail.com (C.-C.K.); zhangyanting329@163.com (Y.Z.); 2 Institute of Geographical Sciences and Natural Resources Research, Chinese Academy of Sciences, Beijing 100101, China; Email: lixb@igsnrr.ac.cn

**Keywords:** ecological land, land use, cellular automation model, ecosystem management

## Abstract

Ecological land is like the “liver” of a city and is very useful to public health. Ecological land change is a spatially dynamic non-linear process under the interaction between natural and anthropogenic factors at different scales. In this study, by setting up natural development scenario, object orientation scenario and ecosystem priority scenario, a Cellular Automation (CA) model has been established to simulate the evolution pattern of ecological land in Beijing in the year 2020. Under the natural development scenario, most of ecological land will be replaced by construction land and crop land. But under the scenarios of object orientation and ecosystem priority, the ecological land area will increase, especially under the scenario of ecosystem priority. When considering the factors such as total area of ecological land, loss of key ecological land and spatial patterns of land use, the scenarios from priority to inferiority are ecosystem priority, object orientation and natural development, so future land management policies in Beijing should be focused on conversion of cropland to forest, wetland protection and prohibition of exploitation of natural protection zones, water source areas and forest parks to maintain the safety of the regional ecosystem.

## 1. Introduction

Land use and cover change (LUCC) have various impacts on global climate change, food safety, soil degradation and biodiversity and thus received much attention from different disciplines [1,2,3,4,5]. In the past 50 years, destruction and fragmentation of natural environment in the World due to agricultural land expansion, urbanization, industrial land expansion and transportation network construction has increased the human footprint on the natural ecosystem [6,7]. Loss of natural space caused by land use change has been the main threat for biodiversity [7,8,9].

With the expansion of urban and industrial land requirements in China, land use with ecological protection are present in the urban, agro-forest and environment protection planning documents, including nature reserves, drinking water sources, country parks, wetland parks and ecological forests. Those land use types are obviously distinguished from city and rural space and are defied by either ecological land or ecological space [10]. Ecological land is like the “liver” of a city, which helps maintain the health of a regional ecosystem and is very useful to public health [11,12]. Recently, some unreasonable land use types due to urbanization and industrialization have threatened ecological land. First, urban expansion has converted plenty of natural land into constructed land impacting the regional ecosystem health. Second, many ecological land types including wetlands and forest lands are faced by agricultural land exploitation [12]. Over-exploitation of ecological land may lead to many eco-environmental problems, including biodiversity loss, ecological degradation and decrease in the ecological regulating ability [13].

Because of the potential value of ecological land and eco-environmental effects of LUCC, it is necessary to explore the quantitative spatial change of ecological land and related evolution mechanisms to understand the spatial strategies of regional eco-safety. Traditionally protection strategies for ecological space still rest mostly on a macro perceptual level and quantitatively explicit demands of eco-space protection are difficult to consider [14]. Pattern regulation models based on the spatially explicit model indeed explore the mechanism of formation of eco-space and reflect the pattern character of horizontal direction impacting on ecological processes in the landscape ecology [15,16]. 

Ecological land change is a spatially dynamic non-linear process involving interactions between natural factors and anthropogenic ones at different scales. Conventional statistical models are insufficient to simulate these effects. The CA model has many advantages for simulating complex space systems and can be a supplement or replacement for some top-down models in certain fields [11,17]. In addition, the CA model is useful and powerful because space and time are interdependent and thus it can simulate geographical spatial patterns [18,19]. Based on these properties, a CA model is used for the description of macro-control of regional ecological land and is able to present the non-linear properties and management strategies that cannot be described by conventional mathematical models. So far many scholars have used CA model in the growth, expansion, land use change and planning simulation of cities [19,20,21,22]. It is now applied to the identification and regulation of protective space and forest protection planning [11,23,24,25,26]. 

Simulation processes of pattern regulation of ecological land under different scenarios by a CA model can test the spatial effects and reliability of protection strategies. Therefore in this study by setting up a natural development scenario, object orientation scenario and ecosystem priority scenario, a CA model has been established to explore the evolution patterns of ecological land in Beijing. By comparing the differences between these scenarios, we are able to discover the spatial effects of different control policies for ecological land and provide guidance to maintain the safety of the regional ecosystem and sustainable development in Beijing.

## 2. Materials and Methods

### 2.1. Study Area

**Figure 1:**
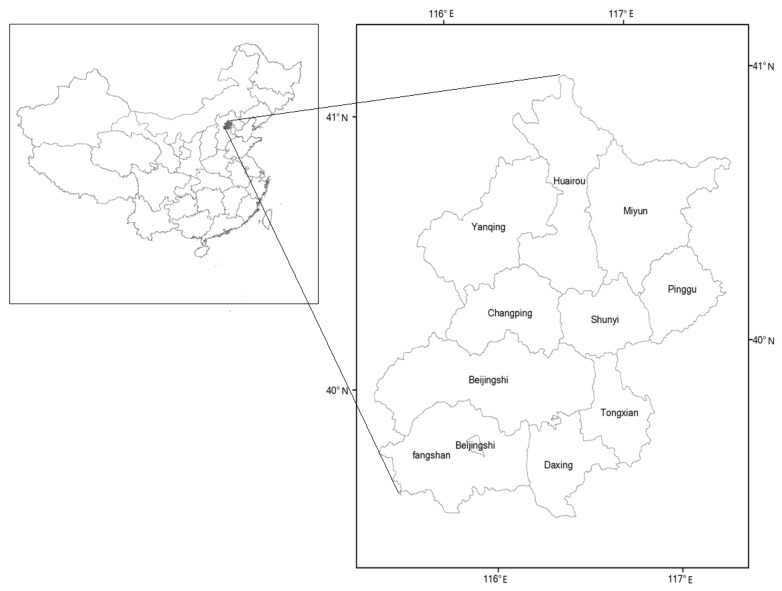
Study area.

The study area (39°38′N–41°05′N) is located in the northern region of China, covering approximately 16,807 km^2^ (Figure 1). 

The area belongs to the continental monsoon climate zone, with an average annual temperature of 14 °C and an average annual rainfall of 484 mm. Average annual sunshine is about 2,000–2,800 hours. The total radiation amount of the annual sunshine is about 112–136 kcal/cm^2^. Its administrative area comprises 14 districts and two countries. The study area has a population of about 16.95 million, with an average population density of 1,033 people/km^2^ [27]. Recently, because of its fast urbanization, natural ecosystems are being increasingly replaced by urbanization. Exploring the evolution mechanism and regulation strategies of ecological land is helpful to public health in Beijing. 

### 2.2. Data

Land use data in this study was derived from 1:100,000 national land use database of Data Center for Resources and Environmental Sciences of Chinese Academy of Sciences (RESDC) which has realized the successive monitoring for every five years since the late 1980s (henceforth referred to as 1990). In this study, land use type was divided into four classes and 12 subclasses (see Table 1). DEM data with a spatial resolution of 30 m × 30 m is also collected from Data Center for Resources and Environmental Sciences of Chinese Academy of Sciences, and then all data were resampled into the 100 m × 100 m. Social-economical data at the county level in this study were derived from the Beijing statistics yearbook from 1990 to 2006.

**Table 1:** Land use and land cover of the study area.

Land use/ land cover class	Land use/land cover subclass
Cultivated land	Paddy field
	Dry field
Ecological land	Forest
	Grassland
	Wetland
Construct land	City or town region
	Village residential area
	Rest construct land
Other land	Sand land
	Bare ground
	Bare rock
	Rest of used land

At present, most research on the CA model focuses on the choices of location factors, such as distance to the nearest urban center, roads, *etc*., which lack the consideration of socio-economic factors that play important roles in land use change due to its difficulty for spatial quantification. However, these factors must not be ignored because of their significant impacts on the land-use systems. To be more realistic when carrying out the CA model, we take two socio-economic factors (population and GDP) into account based on the natural factors. Eventually, we select five natural variables and two social variables (see Table 2) based on related research results. The original attribute values are standardized to (0, 1), and interpolated to a 100 m × 100 m data grid. In this study, the acquisition and pretreatment of geographic spatial data is based on the ArcGIS9.3 software, and the pretreatment of remote sensing image is based on ERDAS software. The model is developed and realized under the Visual Basic environment.

**Table 2:** Spatial variables needed by finding translation rules for the logistic regression model.

Variable types		Acquisition method	Normalized value
Dependent variables	Convert to urban land between 2000 and 2005	Overlay analysis of ArcGIS9.3	0–1
	Convert to cultivated land between 2000 and 2005	Overlay analysis of ArcGIS9.3	0–1
	Convert to ecological land between 2000 and 2005	Overlay analysis of ArcGIS9.3	0–1
Independent variables of natural factors	Distance to the nearest town center	Eucdistance function of ArcGIS9.3	0–1
	Distance to the nearest highway	Eucdistance function of ArcGIS9.3	0–1
	Distance to the nearest river	Eucdistance function of ArcGIS9.3	0–1
	DEM	Digitalization of relief map	0–1
	Slope	Surface analysis module of ArcGIS9.3	0–1
Independent variables of social factors	*Per capita* GDP	Inverse Distance Weight’s Interpolation of ArcGIS9.3	0–1
	Population density	Inverse Distance Weight’s Interpolation of ArcGIS9.3	0–1

### 2.3. Methods

#### 2.3.1. Scenario Setup

Due to the uncertainty of future management policy for land use development, we set up three scenarios to simulate the patterns of ecological land: 

(1) Scenario I, natural development scenario

This scenario assumes that the change of regional ecological land is based on the past trend of land use change and economic drivers can be used to predict the spatial development of ecological land use in 2020. 

(2) Scenario II, object orientation scenario

This scenario assumes that the change of regional ecological land, based on the designed development path and environmental protection objective, which involves constraints of future construction land requirement and cropland protection, can be used to predict the spatial development of ecological land use in 2020.

(3) Scenario III, ecosystem priority scenario

This scenario assumes that key ecological land (with important ecosystem service) should be protected with priority and then we consider the natural environmental characteristics and land use planning to control urbanization, economic development, land use expansion and cropland development in order to predict the spatial development of ecological land use in 2020.

#### 2.3.2. Framework of CA Model

**Figure 2:**
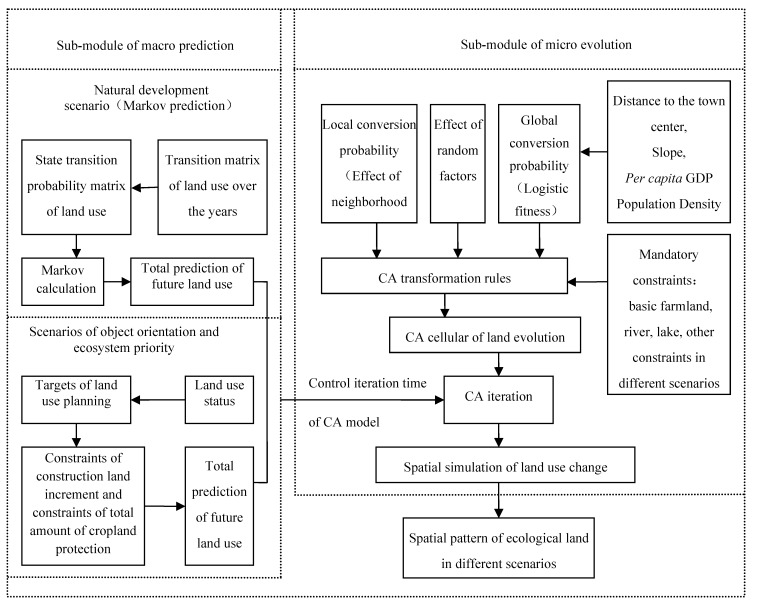
Framework of CA model for ecological land regulation under different scenarios.

The CA model consists of two sub-modules, macro prediction and micro evolution pattern. Figure 2 presents the framework of the model.

The framework of the model is from the viewpoint of solving the defects of traditional CA in spatial simulation. First, we set up three scenarios for land use patterns, (*i.e.*, natural development, object orientation and ecosystem priority) while the government’s planning objectives are embedded in scenarios of object orientation and ecosystem priority. Each scenario is used to predict the quantity of each land use type in the future and control the iteration time of the CA model. Second, based on the CA model, we substitute the complex formulation of transformation rules with cellular synthesis conversion probability after introducing stochastic components by choosing the natural and social factors that influence land use change, calculating the cellular fitness of logistic regression and confirming the effects of domain and constraints. Finally, by using GIS we simulate the scenario variation of regional spatially land use change with the general model. 

#### 2.3.3. Sub-Module of Macro Prediction

(1) Macro prediction of regional land use in a natural development scenario

The development direction of each land use change is bilateral, which means it can be transferred from a current one to others. In such a random process of transformation, type, quantity, degree and form of land use change are constant. This kind of process, which cannot be interpreted by mathematics, includes several conditions that satisfies Markov's framework. Since land use change has the properties of a Markov process, we can use Markov chains to predict the amount of land use while keep land policy remains stationary. The model is interpreted as: 


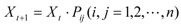
(1)

where *X_t+1_* and *X_t_* represent the system state of land use at time *t* and *t*+1, respectively; *P_ij_* is the matrix of the state transition probability in different periods. We can construct the Markov state transition probability matrix by counting the regional land use transition matrix in different periods. When the number of cell iterate to the range of the Markov predictive value (*X_t+1_–N*, *X_t+1_+N*) (*N* is the threshold value), model iterations stop. The provision is set to solve the problem of determining the time in traditional CA model. 

(2) Macro prediction of regional land use in the object orientation and ecosystem priority scenarios

The macro prediction of regional land use in the scenarios of object orientation and ecosystem priority can predict the amount of construction land and cultivated land. Then we predict the total amount of ecological land (both used and unused land), based on the constraints of construction land increment and total amount of cultivated land protection confined in the regional land use planning and basic data. 

#### 2.3.4. Sub-Module of Micro Evolution Pattern

There are five basic components of CA including cellular (C), state (S), time (T), neighborhood (N) and transformation rules (R). The CA model is regarded as composed by a cellular space and the transfer function defined in the space (that is, *S*_(*t*+1)_ = f(*S*_(*t*)_,*N*)). This study proposes the cellular fitness calculation based on logistic regression, and uses the neighborhood function to determine the influence of the cellular domain space. Finally, we use the confined conditions to stochastically calculate the transition probability of CA so as to tackle the problems that the traditional transition rules cannot verify.

##### 2.3.4.1. Determination of Global Conversion Probability

In the process of land use development simulation, the higher the probability of a cell converting to other land use types, the greater the suitability of land converted into the other land use. A series of spatial variables affecting the land use development can be used to measure the suitability of land use development. These variables include the natural and socio-economic factors in the land evolution system. This study constructs the CA cellular fitness calculation based on the related literature [16] of Logistic regression model. The formula is given as:


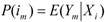
(2)

where *P*( *i_m_*) represents the probability of selecting the type *m* of land use when land unit *i* in state *X_i_*; *X_i_* is the impact factor of land use change, such as distance to the town center, distance to a river and population density; *m*∈{cultivated land, construction land, ecological land, other land}.

After using Theil normalization on the logistic regression equations, conversion rate of land use type can be expressed as:


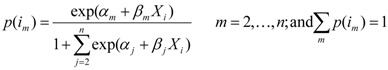
(3)

By solving the equations, the probability sets of cellular unit *i* transferred from the original land-use type to land-use type *j* in a period can be obtained. The maximum probability corresponding to each cell is the land use type that the cell could be transferred to in the next period. The study adopts the cellular fitness of land as CA global conversion probability, *P*( *i_j_*) values in (0–1).

##### 2.3.4.2. Determination of Local Conversion Probability

The unit development probability *P_ij _*takes the various spatial distance variables into consideration. However, the effect of neighborhood is an important factor in CA. Therefore, we need to consider the impact of neighborhood on the central unit by adding a dynamic factor to make land use more intensive in CA and prevent messy phenomena of spatial distribution. The formula is given as:


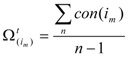
(4)

where 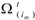 stands for the local probability that block units *i* suits for developing land use type *m* at time *t*; *con(i_m_)* is the total number of the pixels neighborhood {cultivated land, construction land, ecological land, other land}, and *n* is the total number of pixels within a neighborhood.

##### 2.3.4.3. The Setting of Mandatory Constraints

The unit constraint variables must be taken into account in the CA model. For example, the probability of a water body and basic farmland being transformed into construction land is generally low. Therefore, it is necessary to introduce the unit constraints 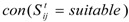 into the CA model (*con* values in (0–1)). In this study, we set different constraints in different scenarios to reflect the degree of protection of regulation on the key ecological land. Specifically, we divide constraints into: (1) For the natural development scenario, the constraint is to prohibit basic farmland evolution; (2) For the object orientation scenario, the constraint is to prohibit water and basic farmland evolution; (3) For the ecosystem priority scenario, its constraints are to prohibit the evolution of core areas and the construction of mountains above 25° and all ecological land. The core area includes basic farmland, water bodies, the first one-level water source protected area, nature reserves, scenic spots and geological parks.

##### 2.3.4.4. Setting up Random Factors

The expansion of land space is influenced and intervened by political factors, human factors, random factors and accidents. Therefore, in order to make the result of the model’s operation closer to the actual situation, we introduce stochastic components into the CA model to reflect the uncertainty existing in the land use system. The stochastic components can be expressed as [28]:


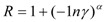
(5)

where γ is the stochastic number values in (0, 1); α is the parameter controlling the impact of stochastic variables with the range and is an integer between 1 and 10.

##### 2.3.4.5. Determination of Synthesis Conversion Probability

By synthesizing the impact of global development probability, conversion probability of local neighborhood and unit constraints and the factors of stochastic component, the development probability of any unit at time t+1 can be expressed as:


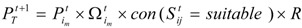
(6)

where 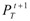 represents the synthetic probability value of land development at time t+1; 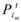 is the probability value of global development of a cellular unit; 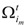 is the probability value of a cellular unit affected by the spatial scope of neighborhood; 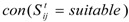 is the constrained values of a cellular unit; *R* is the stochastic variable in the process of land development.

After normalizing the overall probability to the value between (0, 1), we compare the normalized probability with the selected threshold *P_threshold_*:


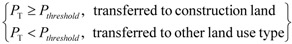
(7)

When *P*_T_ < *P_threshold_* the land is converted to other land use types, *i.e.*, one of {cultivated land, ecological land, other land}. The definition of transformation rules is: in local constraints, we count the number of pixels of {cultivated land, ecological land, other land} within the neighborhood of the current cell, calculate the conversion probability of land use type based on Equation (8). However, in the case of global conversion probability and with the random factors unchanged, we need to calculate the conversion probability of the three {cultivated land, ecological land, other land} land use types separately and take the maximum, if:


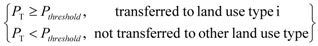
(8)

where land use type *i* is one of {cultivated land, ecological land, other land}.

## 3. Results and Discussion

The transition change of different land use types in Beijing from 2000 to 2005 is listed in Table 3. From this Table, we see that the area of ecological land occupied by cultivated land and construction land is 876.13 km^2^ and 305.07 km^2^, respectively. About 20% of cultivated land is replaced by construction land from 2000 to 2005 in this study area. Total ecological land decreases 4.5% and construction land increased 34.78% in the past five years, indicating that loss of ecological land is mainly caused by urbanization. Main ecological land types, including grass land and wetland, were exploited by agriculture land in Beijing because of the trend to recover from the loss of cultivated land from 2000 to 2005.

**Table 3:** Transition matrix of land use change from 2000 to 2005 in Beijing (km^2^).

2005	Cultivated land	Ecological land	Construction land	Other land	Total area
2000					
Cultivated land	3,012.09	618.74	914.19	8.83	4,553.85
Ecological land	876.13	8,476.67	305.07	16.43	9674.3
Construction land	342.4	128.64	1,664.36	5.29	2,140.69
Other land	2.04	13.1	1.7	0.04	16.88
Total area	4,232.66	9,237.15	2,885.32	30.59	16,385.72

**Figure 3:**
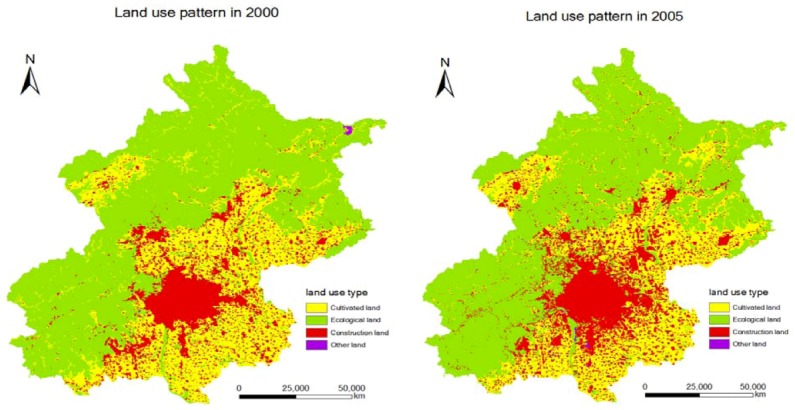
Change of land use pattern from 2000 to 2005 in Beijing.

Figure 3 reflects the spatial change of land use patterns from 2000 to 2005 in Beijing. From Figure 3, we see that spaces near the urban space are replaced by construction land. 

This means that the closer we are to a town or city, the more likely it is that ecological land or cultivated land will be converted to construction land. The expanding variables of land use types from 2000 to 2005, as dependent variable *y*, are overlaid on the influence factor graph (independent variable *X_i_*). The data of random sampling is taken as the proportion of 20%. The point coordinates of random points are obtained by randomized algorithm of the Random function provided by Visual Basic language, and saved as ASCII format files. Next we introduce the sample data into SPSS software to perform the logistic regression model, and calculate the cellular fitness according to the logistic regression model. We use 3 × 3 neighborhood to analyze the effects of neighborhood space. The mandatory constraint of natural development scenario is basic farmland protection, which is assigned to 0. The Markov total prediction was obtained by calculating the transition matrix of land use over the years and controlling the number of CA iterations.

**Figure 4:**
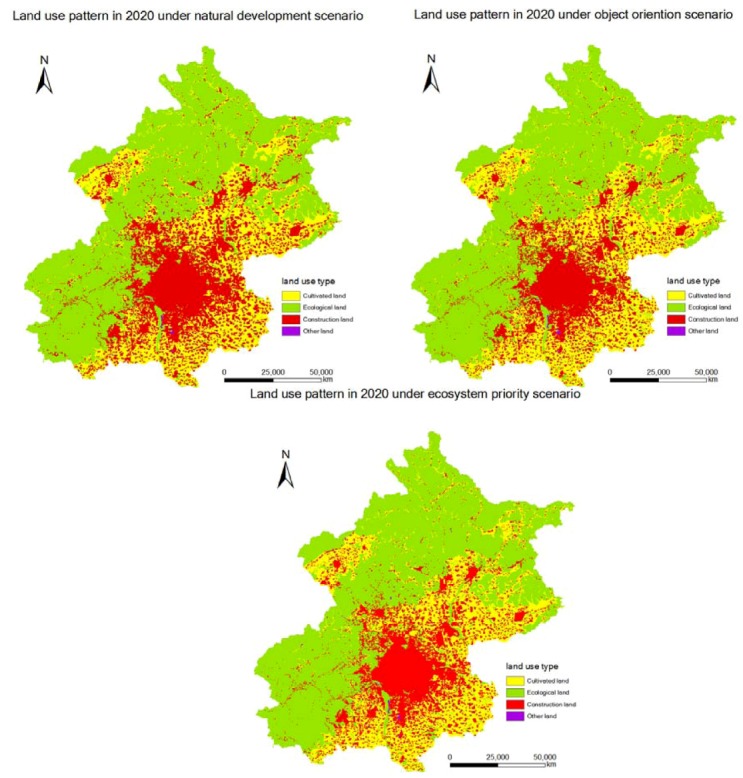
Development pattern of land use in 2020 of Beijing under different scenarios.

We choose the land use type in 2000 as basis to simulate the development of land use in 2008 and obtain the model parameters. Meanwhile, we use overlay analysis of ArcGIS9.3 to test the precision of each point. That is, we calculate the ratio of cellular number, with inconsistent land type and the total amount of cells. The general precision we achieved is 82.36% and the simulation precision seems good. By changing constraints, we next simulated the development patterns of land use in 2020 in scenarios of natural development, object orientation and ecosystem priority (Figure 4, Table 4).

From Figure 4 and Table 4, we conclude that in the land use of the natural development scenario in Beijing with the development of the current trend without regulation, the predicted construction land increased by 32.94% in 2020 while cultivated land decreased by 10.26% and ecological land decreased by 509.02 km^2^. In accordance with the current trend without controlling the scale of the urban and rural construction land, the cultivated land reduction is too large. There has a gap between the cultivated land quantity determined in land use planning in 2020 when ecological land is replaced by construction land and cultivated land because of the urban development and land exploitation of cultivated land.

**Table 4:** The predicted areas of land use in 2020 of Beijing in different situation.

Land usetypes	Land use status (2005)		Natural development scenario (2020)		Object orientation scenario (2020)		Ecosystem priority scenario (2020)	
	Area (km^2^)	Proportion (%)	Area (km^2^)	Proportion (%)	Area (km^2^)	Proportion (%)	Area (km^2^)	Proportion (%)
Cultivated land	4,232.66	25.83	3,798.57	23.18	4,003.17	24.43	3,903.64	23.82
Ecological land	9,237.15	56.37	8,728.13	53.27	9,105.78	55.57	9,266.64	56.55
Construction land	2,885.32	17.61	3,835.82	23.41	3,189.32	19.46	3,189.32	19.46
Other land	30.59	0.19	23.20	0.14	29.15	0.18	25.72	0.16
Total area	16,385.72	100	16,385.72	100	16,385.72	100	16,385.72	100

According to the land use planning (2006–2020) in Beijing, the increment of urban and rural construction land should not exceed 304 km^2^, and the decrease of cultivated land from 2006 to 2020 should be controlled at 7.97%. Table 4 shows the results of land use development. Under the object orientation scenario, the simulation and prediction result of construction land, cultivated land and ecological land in 2020 changed by 10.54%, -5.42% and -131.37 km^2^, respectively.

From Table 4, we can conclude that under the ecosystem priority scenario, the simulation and prediction result of land use development in 2020 not only meet the control object for construction land and basic farmland, but also increased the area of ecological land by 29.49 km^2^, so from the ecosystem protection point of view, these scenarios could be arranged as ecosystem priority scenario > object orientation scenario > natural development scenario.

We use the Fragstats3.3 software to calculate the land use pattern index for different scenarios, and overlay the simulated land use pattern map in 2020 on related research results. We analyze the occupation of key ecological land under different scenarios to compare the protective effects of ecological land in different scenarios. The concrete results are shown in Table 5.

**Table 5:** Compare of protective effect of ecological land in different scenarios.

Evaluation index	Status (2005)	Natural development scenario (2020)	Object orientation scenario (2020)	Ecosystem priority scenario (2020)
Loss quantity of key ecological land in low security level (km^2^)	0	311.86	186.32	6.33
Loss quantity of key ecological land in high security level(km^2^)	0	1,567.10	1195.19	1,138.25
Largest patch index (LPI)	48.089	48.771	50.537	51.538
Cohesion index of patch (COHESION)	99.915	99.908	99.903	99.909
Splitting index (SPLIT)	4.322	4.202	3.913	3.763
Aggregation index (AI)	96.243	96.157	96.090	96.251

In Table 5, from the area occupied by ecological land point of view, these scenarios could be arranged as (the loss of) the ecological land under natural development scenario > object orientation scenario > ecosystem priority scenario. Therefore, the study area should give priority to ecological controlling measures, strictly limiting the development and construction of restricted zones, implement a policy of returning farmland to forest or grasslands, protect the region's key ecological land and maintain the regional ecological security. 

Largest patch index (LPI) is equal to the percentage of the total landscape that is made up by the larger patch, which may reflect the degree of lager patch influencing on the landscape. The cohesion index of patch (COHESION) was calculated as an estimate of connectivity. Both are measures of landscape dominance. From the landscape dominance, the largest patch index of ecological land under the ecosystem priority scenario is the largest, which is 51.538, followed by the object orientation scenario—50.537. The results show that the ecosystem priority scenario effectively protects the dominant patch of ecological land and is able to maintain the ecological security of the entire landscape.

Aggregation index (AI) reflects the degree of aggregation of different patch types in the total landscape. Splitting index is related to another index called the degree of division index, which is a measure of aggregation within a landscape. The splitting index reflects the degree of dispersion of patches in the spatial distribution. The aggregation degree of ecological land under the ecosystem priority scenario is the largest (96.251), followed by the natural development and object orientation scenarios. The results illustrate that the ecological land patches tend to be more clustered in the ecosystem priority scenario. In terms of the splitting index of ecological land, we can show the ranking of scenarios as natural development scenario > object orientation scenario > ecosystem priority scenario. The ranking illustrates that the ecological land patches tend to be more dispersed in the natural development scenario.

Considering three factors such as the total area of ecological land, the loss of key ecological land and the ecological land pattern in different scenarios, we could arrange the scenarios as the ecosystem priority scenario > object orientation scenario > natural development scenario.

Multi-Agent System (MAS) is an important tool to analyze and study the decision-making behavior in LUCC [29,30]. The “inherent” intelligence, adaptability, interactivity and initiative of MAS are particularly suitable to simulate a variety of policies and individual decisions, the optimal decision for the sustainable use of natural resources management, and provide the optimal choice for the sustainable utilization management of natural resources. It will be the developing trend of the CA model in the study of land-use systems, as well as the aim of this study. By combining the CA model of ecological land evolution proposed in this study with the multi-agent model and establishing a new model, we can effectively simulate ecological land evolution under the combined effects of decision-making behaviors of multi-agents (government, developers and farmers).

## 4. Conclusions

Ecological land is very helpful to improve our health and maintain regional eco-security. Using a scenario analysis, this study establishes a CA model of land use evolution to simulate the land use development patterns of Beijing in 2020 by setting up scenarios of natural development, object orientation and ecosystem priority. The main results are as follows:

From the amount of each land use type, construction land increased by 32.94% for the natural development scenario, breaking the scale of urban and rural construction land in 2020 in the planning project. Cultivated land area is reduced by 10.26%, which represents a big gap from the arable land quantity determined in land use planning for 2020. Meanwhile, the ecological land was occupied as a result of the development of construction land and cultivated land. For the object orientation and ecosystem priority scenarios, both indices of construction land and cultivated land meet the requirements of land use planning. However in the ecosystem priority scenario, there exists an increasing trend for the ecological land in 2020. In terms of the loss of key ecological land, these scenarios could be arranged as natural development scenario > object orientation scenario > ecosystem priority scenario.

From the space form of ecological land, the simulation scenario of ecosystem priority can effectively protect the dominant patches of ecological land. At the same time, the aggregation degree between dominant patches of ecological land is high. When considering the amount of ecological land area, the quantity of loss of key ecological land and the spatial form of ecological land, the ecological land pattern in different scenarios in 2020 could be arranged as the ecosystem priority scenario > the object orientation scenario > the natural development scenario.

In this paper, three scenarios for simulating the evolution of ecological land are established to reflect different policies for protecting ecological land. Modeling results show that the ecosystem priority scenario is best, which means future land management policies should be focused on this aspect. Those results are meaningful to implement effective environment management.

Some preferred policies should be carried out in the future land use management in Beijing, including the policy of returning farmland to forest or grasslands, wetland protection and prohibiting the exploitation of restricted zones to maintain the health and safety of the regional ecosystem. The restricted zones include first one-level water source protected areas, nature reserves, the scenic spots and geological parks.

## References

[1] Lambin E.F., Turner B.L., Geist H. (2001). Our emerging understanding of the causes of land use and cover change. Global. Environ. Change..

[2] Li X.B. (1996). Core of Global environmental change research: Frontier in land use and coverage change. Acta Geo. Sin..

[3] Vitousek P.M. (1997). Human domination of Earth’s ecosystems. Science.

[4] Xie H.L., Liu L.M., Li B., Zhang X.S. (2006). Spatial autocorrelation analysis of multi-scale land-use changes: A case study in Ongniud Banner, Inner Mongolia. Acta Geo. Sin..

[5] Schipper J., Chanson J.S., Chiozza F., Neil A., Cox N.A., Hoffmann M., Katariya V., Lamoreux J., Rodrigues A.S.L., Stuart S.N. (2008). The status of the world’s land and marine mammals: Diversity, threat, and knowledge. Science.

[6] Sanderson E.W., Jaiteh M., Levy M.A., Redford K.H., Wannebo A.V., Woolmer G. (2002). The human footprint and the last of the wild. Bioscience.

[7] Vimal R., Pluvinet P., Sacca C., Mazagol P.O., Etlicher B., Thompson J.D. (2012). Exploring spatial patterns of vulnerability for diverse biodiversity descriptors in regional conservation planning. J. Environ. Manage..

[8] Sala O.E., Chapin F.S., Armesto J.J., Berlow E., Bloomfield J., Dirzo R., Huber-Sanwald E., Huenneke L.F., Jackson R.B., Kinzig A. (2000). Global biodiversity scenarios for the year 2100. Science.

[9] Wilcove D.S., Rothstein D., Dubow J., Phillips A., Losos E. (1998). Quantifying threats to imperiled species in the United States. Bioscience.

[10] Xie H.L., Li X.B. (2011). A method for identifying spatial structure of regional critical ecological land based on GIS. Resour. Sci..

[11] Mitsova D., Shuster W., Wang X.H. (2011). A cellular automata model of land cover change to integrate urban growth with open space conservation. Landsc. Urban Plan..

[12] Xie H.L. (2011). Analysis of regionally ecological land use and its influencing factors based on a logistic regression model in the Beijing–Tianjin–Hebei region, China. Resour. Sci..

[13] Brooks T.M., Mittermeier R.A., da Fonseca G.A.B., Gerlach J., Hoffmann M., Lamoreux J.F., Mittermeier C.G., Pilgrim J.D., Rodrigues A.S.L. (2006). Global biodiversity conservation priorities. Science.

[14] Byomkesh T., Nakagoshi N., Dewan A.M. (2012). Urbanization and green space dynamics in Greater Dhaka, Bangladesh. Landsc. Ecol. Eng..

[15] Orsi F., Church R.L., Geneletti D. (2011). Restoring forest landscapes for biodiversity conservation and rural livelihoods: A spatial optimisation model. Environ. Modell. Softw..

[16] Wu J.G., Hobbs R. (2002). Key issues and research priorities in landscape ecology: An idiosyncratic synthesis. Landsc. Ecol..

[17] Couclelis H. (1997). From cellular automata to urban models: New principles for model development and implementation. Environ. Plan. B Plan. Des..

[18] Zhou C., Sun Z., Xie Y. (1999). Geographical Cellular Automation Research.

[19] Li X., Yeh A.G.O. (2000). Modelling sustainable urban development by the integration of constrained cellular automata and GIS. Int. J. Geogr. Inf. Sci..

[20] Clarke K.C., Gaydos L.J. (1998). Loose-coupling a cellular automaton model and GIS: Long-term urban growth prediction for San Francisco and Washington/Baltimore. Int. J. Geogr. Inf. Sci..

[21] Mahiny A.S., Gholamalifard M. (2007). Dynamic spatial modeling of urban growth through cellular automata in a GIS environment. Int. J. Environ. Res..

[22] Liu X.P., Li X., Shi X., Zhang X.H., Chen Y.M. (2010). Simulating land-use dynamics under planning policies by integrating artificial immune systems with cellular automata. Int. J. Geogr. Inf. Sci..

[23] Mondal P., Southworth J. (2010). Evaluation of conservation interventions using a cellular automata-Markov model. For. Ecol. Manage..

[24] Tattoni C., Ciolli M., Ferretti F. (2011). The fate of priority areas for conservation in protected areas: A fine-scale Markov chain approach. Environ. Manage..

[25] Mathey A.H., Krcmar E., Tait D., Vertinsky I., Innes J. (2007). Forest planning using co-evolutionary cellular automata. For. Ecol. Manage..

[26] Mathey A.H., Krcmar E., Dragicevic S., Vertinsky I. (2008). An object-oriented cellular automata model for forest planning problems. Ecol. Model..

[27] Zhang B.A., Xie G.D., Zhang C.Q., Zhang J. (2012). The economic benefits of rainwater-runoff reduction by urban green spaces: A case study in Beijing, China. J. Environ. Manage..

[28] White R., Engelen G. (1993). Cellular-automata and fractal urban form—A cellular modeling approch to the evolution of urban land-use patterns. Environ. Plan. A.

[29] Zhang H.H., Zeng Y.N., Bian L. (2010). Simulating multi-objective spatial optimization allocation of land use based on the integration of multi-agent system and genetic algorithm. Int. J. Environ. Res..

[30] Le Q.B., Park S.J., Vlek P.L.G. (2010). Land Use Dynamic Simulator (LUDAS): A multi-agent system model for simulating spatio-temporal dynamics of coupled human-landscape system 2. Scenario-based application for impact assessment of land-use policies. Ecol. Inform..

